# Narratives of climate adaptation and linkages to psychosocial and nutritional health in a Zimbabwean rural community

**DOI:** 10.1016/j.wds.2025.100205

**Published:** 2025-06

**Authors:** Sandra Bhatasara, Chijoke Nwosu, Lesley Macheka, Admire M. Nyamwanza

**Affiliations:** a Environment Climate and Sustainable Development Institute, University of Zimbabwe, 630 Churchill Avenue, Mt Pleasant, Harare, Zimbabwe; b Department of Economics and Finance , University of the Free State, 205 Nelson Mandela Drive, Park West, Bloemfontein, South Africa; c Marondera University of Agricultural Sciences and Technology Innovation and Industrialisation P.O Box 35, Marondera, Zimbabwe; d Department of Economic Performance and Development, Human Sciences Research Council 116-118 Buitengrancht Street Cape Town 8801, South Africa

**Keywords:** Climate change, Adaptation, Adaptation impacts, Nutrition, Psychosocial

## Abstract

In the face of unprecedented climate change, adaptation has emerged as important for communities and nations to deal with the devastating effects of the phenomenon. It is inevitable that communities must adapt, although evidence in several regions, including Zimbabwe also point towards maladaptation. A plethora of studies have been developed to understand adaptation practices and processes, including the impacts of various adaptive strategies. However, this approach has been limited to particular fields such as livelihoods studies, with clear evidence in Zimbabwe that heath issues vis-à-vis adaptation outcomes have not been taken into consideration at policy, development and research levels. Our study is therefore breaking new research frontiers by exploring the nexus between adaptation strategies and psychosocial and nutritional health outcomes. As an important learning research process into a field where virtually no literature exists in the country, the results are both complex and intriguing. This qualitative study shows positive nutrition benefits such as improved dietary diversity and boost in self –esteem and, improved stress level over food availability as psychosocial health benefits.

## 1 Introduction

This paper seeks to understand the linkages between climate adaptation and psychosocial and nutritional health. Rural communities have been heavily burdened by the changes in climate because they largely depend on ecosystems. The speed of contemporary climate change and variability is feared to transcend the limits of adaptation in many parts of the world. Climate change and variability introduce new challenges in Africa, not only because of the expected rise in temperature and decrease in rainfall, but also owing to the prevailing context of failure to address even non-climatic problems [1]. Thriving human societies are characterised by their adaptability, as attested throughout human existence. The IPCC [2] defines adaptation to climate change as the adjustment in natural or human systems in response to actual or potential climatic stimuli or their effects, which moderates harm or exploits advantageous opportunities. Adaptation to climate change is not just about reducing the incidence and impact of disasters, it involves putting in place measures that will protect humanity from the everyday effects of the climatic changes that are likely to occur [3]. Various types of adaptation can be distinguished, including anticipatory and reactive adaptation, private and public adaptation, and autonomous and planned adaptation [4]. In addition, adaptation action has the potential to reduce adverse effects of climate change and can often produce immediate ancillary benefits. However, it will not prevent all damages.

In Africa, rural households have adapted to such extreme weather events as droughts for decades using varied ways, among them indigenous knowledge. In some extremely dry parts of the Sahelian region, households have even moved beyond dependence on climate. This means households are diversifying their livelihoods beyond on-farm activities that are dependent on climate to other non-farm activities such as wage employment, petty trading, seasonal labour jobs and migration. Some scholars demonstrated how agro-pastoralists in Kenya used indigenous knowledge to monitor, mitigate and adapt to drought [5]. Similarly, in Burkina Faso, Roncoli et al. [6] discussed how farmers utilise traditional forecasting knowledge to formulate seasonal expectations. In northeast Nigeria, Mortimore and Adams [7] divulged how traditional adaptive strategies have evolved in response to crises in rainfall. And, in southern Africa, studies by Thomas et al. [8] Ziervogel and Calder [9] and Ziervogel et al. [10] among others have investigated local adaptation strategies to climate change and variability.

Importantly, adaptation represents a pragmatic means of achieving sustainable development in the longer term. In this case, adaptation is conceived within the context of multiple stressors and vulnerabilities, and with concern for intra- and inter-generational equity, environmental integrity and poverty eradication [1]. It becomes a paradigm to guide long term development successfully in view of increased risk from climatic, social and economic change. Adaptation presents the opportunity to ‘adapt forward’ and work along an aspired development pathway towards the Sustainable Development Goals instead of regressing to the state of affairs before climate change [11]. Coupled with that, it is now widely realised that adaptation strategies could stimulate harmful effects, conjuring up more complex questions around the aspect of sustainability [12]. It has been long established that adaptation processes can possibly worsen inequalities in well-being by producing winners and losers [13].

Having said the above, whilst various studies have assessed different aspects of climate adaptation actions as bad or good, implementation science is shifting towards understanding adaptations and their impact [14]. Most studies lack constructs to consider “ripple effects” of adaptations (i.e., both intended and unintended impacts on outcomes, recognizing that an adaptation designed to have a positive impact on one outcome may have unintended impacts on other outcomes) (ibid). Specific to this paper, we submit that explicit focus on understanding the health impacts of adaptation actions has not been appreciated, yet community health aspects directly link with livelihood resilience as well as the physical, social and economic well-being of a society. In the same vein, whilst adaptation policy instruments in most countries, (such as the National Climate Policy and the National Climate Change Response Strategy in the case of Zimbabwe), acknowledge the need for strengthening the surveillance of human health under climatic variability and change, they do not proffer strategies for doing that nor do they address the “how” of tracking and evaluating direct health impacts of adaptation actions in different contexts.

## 2 Background to the paper

This paper derives from a research project entitled ‘*Climate adaptation and sustainable rural health outcomes in Southern Africa*’ funded by the Wellcome Trust and implemented from 2019 to 2022. The project sought to assess the complex health impacts of some of the major climate adaptation actions in rural Southern Africa, using case studies of Zimbabwean communities located in the mid-Zambezi Valley area, in the north of the country, along the borders with Zambia and Mozambique. The specific objective was to evaluate the nutritional and psychosocial health impacts of two of some of the major adaptation actions in Southern Africa using case studies of Zimbabwean communities. The climate adaptation actions whose health impacts were evaluated under this project are:

a) Shifting from predominantly maize farming in main dryland fields to the drought-tolerant sorghum crop over the years, and
b) Increased reliance on indigenous knowledge: (a) in wild fruits (during crop failure), and to predict rainfall patterns and droughts using unique meteorological, insect and atmospheric indicators

The key question driving the project was: How have localised climate adaptation actions led to different nutritional and psychosocial health outcomes and what can be done to harness the positive outcomes and address the negative ones for sustained rural household and community resilience? This paper was developed from the results of the aforementioned project. This paper focuses on the following:

a) To determine the nature of the two strategies being used in the communities.
b) To identify the positive and negative nutritional and psychosocial health impacts of the two adaptation actions under focus in the case study area.
c) To proffer recommendations from this study about how identified effective adaptation actions can be effectively upscaled and applied in similar vulnerability contexts in Zimbabwe and in Southern Africa at large.

## 3 Materials and methods

This paper is based on insights from qualitative data collected under the project. Data collection commenced with desk review that continued through the project cycle. This review included an analysis of relevant literature and policy documents on climate variability and change, climate change adaptation, the local health system, the agricultural systems, livelihoods, as well as climate services in the case study area. The research also included field data collection as outlined in the following sub-sections.

### 3.1 Field data collection

#### 3.1.1 Key informant interviews

KIIs were conducted with a select sample of relevant stakeholders at national, provincial and local levels. At the national and provincial levels, stakeholders interviewed included officials from the Department of Climate Change Management, Food and Nutrition Council of Zimbabwe, Ministry of Health and Child Care, National Meteorological Services, the Ministry of Agriculture as well as academic researchers who have worked on issues related to the impact evaluation's aim and objectives in the case study area and in similar contexts in Southern Africa. At the local level, key informant interviews were conducted with local agricultural extension officers, local health officers, traditional leaders, officials from organisations involved in food aid and social support in the area, officers at the local meteorological office as well as heads and senior teachers at local schools. Questions in key informant guides at all levels were informed by all the four research questions.

#### 3.1.2 Stakeholder workshops

Two stakeholder workshops were conducted i.e. one at the national level and another at the sub-national/provincial level, bringing together selected stakeholders to explore issues around the four research questions. The workshops were important in simultaneously collecting ideas and views from different players who hold significant knowledge, insights and experience which informed the impact evaluation.

## 4 Conceptual framework

The project and, subsequently this paper, is guided by an evaluation framework. Evaluation criteria can be thought of as ‘concepts’ that must be addressed in the evaluation [15]. These concepts must be sufficiently defined and must be applied systematically to make evaluative judgments about interventions under focus. Commonly used evaluative criteria include those about equity, gender equality and human rights. Some, used particularly for such development interventions as humanitarian assistance, include such concepts as coverage, coordination, protection and coherence. We used the OECD-DAC criteria [16] commonly used for evaluating development assistance and which have been adopted by most development agencies as standards of good practice in evaluation and impact assessment. These criteria include the following concepts: relevance, effectiveness, efficiency, impact and sustainability. In this paper we use four of the five concepts, guided by what is emerging from the fieldwork findings:

• Relevance: The extent to which the interventions under focus (which are the adaptation actions under scrutiny in the context of this study) are suited to the context i.e. nutritional and psychosocial health needs and priorities of households and communities in the case study area.
• Effectiveness: The extent to which the adaptation actions achieve the (nutritional and psychosocial) outcomes being evaluated.
• Efficiency: How the inputs contributed towards undertaking the adaptation actions e.g. information, technical support etc., translated to outputs.
• Impact: The extent to which adaptation actions under focus have led to differentiated positive and/or negative health outcomes, either directly or indirectly, intended or unintended.
• Sustainability: The extent to which the positive nutritional and psychosocial health impacts realised from the adaptation actions in focus may continue in the medium to long term.

## 5 Contextual setting

**Fig. 1:**
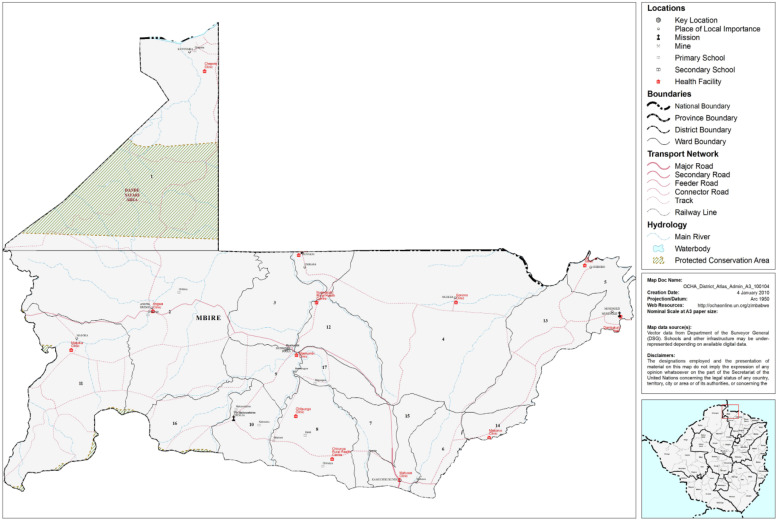
Study site, Mbire district (source; OCHA, 2008) [22].

The study was undertaken in the mid-Zambezi Valley area of northern Zimbabwe, in a district called Mbire in Mashonaland Central Province of the country. The mid-Zambezi Valley is part of the Zimbabwean lowveld and it particularly refers to lands lying north of the Zambezi escarpment bordered by Mozambique to the north and east, and Zambia to the north-west [17]. It consists of an extensive undulating plain averaging 450 m above sea level, descending to 350 m above sea level to the north towards the Zambezi River [18]. Mbire District forms the major part of the low-lying mid-Zambezi Valley in Zimbabwe's Mashonaland Central Province, and it is a semi-arid remote area listed in the country's agro-ecological zones IV and V specifically located 30° 25″ E and 16° 30″S, and encompassing an area of 2700 km^2^s [19]. The district has 17 wards/local administrative geographical boundaries. It is characterised by temperatures of up to 40 °C in summer and low, increasingly irregular rainfalls averaging 450–650 mm annually. There are two clearly defined seasons in the area – a rainy season from December to March and a long dry season from April to November [20]. The mid-Zambezi Valley area presents interesting cases for this evaluation as it is classified among Southern Africa's climate change hotspots [21] (Fig. 1).

### 5.1 Sources of livelihoods

The main sources of livelihoods in the case study area revolve around agricultural activities, particularly crop production and livestock production. There are two crop production systems in the area: upland crop production and riverbank crop production [23]. Upland fields are held by all households in the area and they average 7 to 12 acres per household (ibid). The riverbank crop production system consists of fields along the banks of major rivers, where the majority of villagers hold plots averaging 1 to 5 acres per household (either personal or borrowed). Upland fields consist mainly of shallow sandy clay soils, and are used for dryland crop production involving mainly cotton, the major cash crop in the area, sorghum, maize and groundnut farming.

Livestock raised in the area include cattle, goats, sheep, pigs and poultry – with cattle, goats and poultry forming the majority of animals. Livestock in the area is important as a source of draught power, meat, milk, manure and essential in other important social processes (e.g. paying bride-price, settling serious societal conflicts and payment of fines in traditional courts). As in many other rural African communities, cattle are the most highly ranked form of livestock and the more cattle a household owns, the wealthier and/or financially stable they are (perceived to be) [24]. The number of cattle one has also determines the area that can be planted in upland fields and how fast this can be done, which is important given the fact that timely planting is a major factor in crop success in these marginal areas [25]. It is also a local source of money-making business (e.g. ploughing other villagers’ fields for cash and transporting other people's commodities to the market), as well as spreading social networks because owning more cattle in the area generally means more social recognition and being ‘culturally anchored’ [23].

### 5.2 Vulnerability factors

The study area is beset by a suite of climatic and non-climatic vulnerability factors. The main climate-related vulnerability factors include intra-seasonal dry spells, increased drought cycles and floods. Non-climatic stressors in the area include seasonal malaria, a weak health system, poverty and food insecurity, poor linkage to profitable output markets for agricultural produce, and high wildlife presence making the area marginal for livestock production. These climatic and non-climatic stressors interact in complex ways, impacting negatively on household and community resilience. Since this impact evaluation primarily aims at assessing the health impacts of *climate adaptation actions*, we expand on the discussion around climate-related vulnerability factors in this section so as to get a clear picture and understanding of the nature of climate sensitivities in the study area.

#### 5.1.1 Intra-seasonal dry spells

Previous primary research in the area [23,[26], [27], [28], [29]] reveal that the frequency of dry spells during cropping seasons has increased over the years. These are usually mid-season dry when crops are at a critical stage such as flowering. A season may, therefore, receive normal (or even increased) rainfall amounts; however, these intra-seasonal dry spells, coming at a time when crops are at a critical stage of growth, many a time lead to reduced quantity and quality of crop yields. There have also been shifts in the onset of rains as well as seasons becoming shorter in the area.

#### 5.1.2 Increased drought cycles

Drought cycles have also been increasing in the area. Droughts in the area in recent years have been occurring on average every three years (unlike the five-year average which was usually experienced in the 1980s and 1990s). These droughts have led to total crop failure (especially in upland fields), drying up of rivers and boreholes and livestock deaths. A historical timeline exercise conducted during one research exercise in 2014 to identify serious drought years which have occurred in the area since 1980 showed the following years as having been affected: 1982–1983, 1991–1992, 1994–1995, 2001–2002, 2003–2004, 2006–2007, 2008–2009 and 2012–2013 [27]. The trend clearly showed an increasing frequency in the occurrence of droughts.

#### 5.1.3 Floods

Floods in the mid-Zambezi Valley area have mainly occurred due to two factors: a) Excessive local rains occurring mostly around January/February; and b) Water release from Kariba Dam upstream and backflow from Cabora Bassa Dam in Mozambique further downstream.The second factor arises from the fact that the mid-Zambezi Valley is located downstream of Kariba Dam and upstream of Cabora Bassa Dam. Primary research evidence (obtained by the principal investigator between 2010 and 2017) revealed that flood frequency has increased in the area in the past two decades, with the most recent major flood experience being experienced in 2017.

## 6 Results and discussion

### 6.1 Adaptation practices: relevance, effectiveness, impact and sustainability in Mbire

Previous studies are poignant on the importance of adaptation in local communities to respond not only to climate change stressors but other socio-economic challenges [29,30]. Current qualitative data (key informant interview data) highlights a number of institutional interventions to support adaptation, but our focus is on two community-based adaptations (see 6.1.1, 6.1.2). The sub-sections below outline the strategies that are being utilised at the community and household levels. These are assessed according to the conceptual framework stated earlier.

#### 6.1.1 Switching to drought tolerant crops

Drought tolerant crops are being promoted as a relevant, effective and efficient adaptation strategy to climate change and variability particularly in drought-prone areas like Mbire. In Mbire, farmers have, in recent years, been encouraged to focus on the production of drought resistant crops such as sorghum, cotton and sesame. There has also been a remarkable move from cotton to sorghum farming over the years. Qualitative results show that the most grown crop in Mbire District in recent years is sorghum; grown on an average of 1.64 acres per household and attaining an average yield of 90.87 kg per acre. The growing of the crop resonates with or is relevant to the new climatic conditions. There has also been an increase in the cultivation of sesame and rosella – which are drought tolerant. A key government informant from the Ministry of Agriculture expressed that:

As a ministry we definitely promote drought tolerant crops, and maybe to give you an example, we are currently running or promoting of small grains. In our ministry we call them additional grains, we are looking at the sorghum, we are looking at the pearl millet, and we are looking at the finger millet. We…we actually promoting them, and even if you look at our presidential input packages, these crops were there. We also have sunflower, we also have cowpeas, we are actually working with the, with the World Food Program as a ministry to promote intensively production of small grain crops and cowpeas in marginal districts and for your own information, this season we in 2020 to 2021 season we have upscaled from 13 districts to 30 districts, which are producing small grains, and they are being given inputs by World Food Programme. They are given seeds, we giving them compound D (fertiliser), we are giving the ammonium nitrates for…and we also giving the cowpeas seeds. So, we are definitely promoting.

Traditional cereal grains, namely finger millet, pearl millet and sorghum, and other drought tolerant indigenous vegetables and root crops are not only known to perform effectively than maize under climate shocks, but also have high nutrition potential, particularly for the aged and the sick, pregnant women and children. From existing studies, various institutions have been promoting production of drought tolerant traditional crops, particularly small cereal grains, across the different agro-ecological regions of the country [31,32]. Empirical evidence to date shows that small cereal grains grown on the predominantly sandy soils in Zimbabwe can give reasonable yields of up to 3 tonnes per hectare when well fertilized with mineral and organic fertilizers [33]. There has also been evidence of high nutritional value in finger millet and sorghum grown on smallholder farms and other niche marginal agricultural environments [34].

Linked to this is also the use of traditional variety of seeds suitable for Dande Valley (e.g. use of *kanongo-traditional seed* as compared to hybrids). For sorghum, focus has also been on marcia, then cowpea CBC 1 and 2, which are very popular in the area over the years. Below is an extract from a key informant in the ministry of agriculture:

For the small grains I think integration is directed from the Ministry of Agriculture, I think you can tell that there was a wide scale promotion of the small grains to farmers. They were getting free seeds especially in the dry areas for sorghum and I think maybe if you check the last five years you could see that there was significant jump in terms of production of small grains in those marginal areas. Especially when you consider drought seasons, so the production was quite significant and comparable to other years. We also very high level also under Harvest Plus they are also promoting the orange maize and also the bean variety.

#### 6.1.2 Consumption of wild fruits and vegetables

There is a growing realization that the indigenous knowledge system (IKS) is an effective measure to respond to climate change and ensure a sustainable livelihood. During unforeseen weather events such as droughts and high temperatures, local communities, and households with fewer sources of food rely on wild edible fruits to supplement their diets. From the qualitative study in Mbire District, households consume diverse wild fruits. It was noted by a key informant that:

Yes we have wild fruits called masawu, we have so much of masawu in the valley..there is no other place you will find this fruit other than here, we have too much of it here. There is also mawuyu from the (baobab tree) here in the valley. Ah these fruits l have mentioned are some of the fruits that assist us during drought when they reduce number of meals per day. In the afternoon they can eat masawu or mawuyu……. They can dry masau and they can eat for the whole year.

Studies that speak to the consumption of wild edible fruits in Southern Africa, particularly in Zimbabwe have emerged. They state that most indigenous fruits are eaten fresh, but other rural households also dried and stored selected species for future consumption [35]. Other scholars have documented over 60 wild edible plant species of critical importance in livelihood and survival strategies for rural households and communities in Nhema Communal Area in the Midland Province of Zimbabwe, while others recorded a total of 54 edible fruit species across different villages [35,36].

#### 6.1.3 Use of drought and rainfall indicators

In Mbire, qualitative study results also reveal that the use of indigenous knowledge in predicting forthcoming drought seasons is also a common relevant, effective and efficient adaptive strategy. For example, farmers observe if certain wild fruits are in abundance in the forest, or whether they are scarce. They also observe wind direction, plant phenology, and the behaviour of animals and birds. From the qualitative fieldwork, it was noted by a key informant that:

Yeah I think I can attest that we have variety of indigenous knowledge with regards to rainfall prediction or drought prediction in our societies and I think it is quite common that when you get to a society and you say ’how do you know that this year is a drought year‘, they actually tell you that if the wind is coming from this side or if the clouds are coming from this side this year we are not going to have rainfall, and I think that clear prediction of rainfall or drought also gives evidence of the advice to what these farmers will also put into their field which is quite important to me, then definitely I think there are other methods that they look at…

These predictions effectively support farmers in their cropping activities. Avillage head commented that:

Another indigenous means of weather prediction involves observing heat trends towards October (beginning of the planting season). For example, if the heat trends are such that from June to October there is a steady increase (without some cold spell in-between), then that means a dry season will be beckoning. They also look at wind patterns as connected with how they pan out in certain months particularly between August and October. The flowering of certain trees also assists in showing whether rains are near or not*.*

These predictions support farmers in cropping activities. However, further to the use of local and rainfall and drought indicators, it also emerged at the national level (from key informant interviews and stakeholder workshops) that trust in IKS forecast is not equally shared, so overall their importance was not equally perceived - showing the perceived efficiency and effectiveness of IKS as compared to other knowledge systems. The use of these indicators for weather forecasting was not unilateral, but one could use a combination of indicators in trying to gain greater accuracy [37].

All the same the combination of plants, animals, insects and meteorological and astronomical indicators is used to predict and interpret weather and climate in local communities across Arica. Moreover, the condition of trees, flowering and subsequent bearing of fruits and behaviours of birds, animals and insects is used as an effective warning to looming drought or rainy season [38,39]). This affirms that the Shona people still rely on certain phenomena like trees, birds, frogs, animals, amphibians, insects, grass, wind, moon, lightening and the sun to make reasonable and sometimes fairlyaccurate weather forecasts. Other studies conducted in Zimbabwe, revealed that communities use a number of seasonal indicators to include an abundance of wild fruits like *hacha* (Perinari capensis) or *mazhanje* (Uapaca kirkiana); early flowering of *msasa* (Brachystegia spiciformis) or *munhondo* (Julbenardia globiflora) trees; prevalence and singing of certain birds; and falling of dew in the morning following the first rains [40].

### 6.2 Adaptation actions and household nutritional and psychosocial health outcomes

#### 6.2.1 Nutritional health

The benefits of switching to drought tolerant crops are diverse. For instance, the production of drought resistant crops such as sorghum, does not only guarantee food availability but improved health. On the benefits, one village head in Ward 12 noted that “Basing with previous farming seasons, small grains production seemed to have reduced hunger as compared to maize production. Another village head expressed that “Basing with previous farming seasons, small grains production seemed to have reduced hunger as compared to maize production”. One school headmaster in the district also noted that:

Yeah yes, here we have what we call *masawu* if you ever heard of them or if you ever come across them in Harare. This fruit is found here in large quantities so after harvesting them they dry them, then preserve and make a drink called *maheu*. So the children will then carry them to school though some eat them raw. So it is something that assists the parents when having food challenges.

In terms of health needs, a researcher at a tertiary institution added that:

The only good I can say, for example because they (small grains) are very low in starch, they are very good for like diabetes because they won't raise above sugar level, but otherwise in terms of nutrients, the net intake is quite poor”

Foods from forests are important for peoples’ diets in many countries [41]. The main benefit of consuming wild fruits, insects and vegetables was food security. This was followed by diversified food diets and improved nutrition. The fruits are considered as effective in meeting household nutrition and health needs. Nutritionists in Mbire district noted that some wild fruits (e.g. *mawuyu-baobab fruit*) seem to have nutritional benefits. The majority of those who consume wild edible fruits emphasise the nutritional value besides the monetary value. Consumption of wild fruits was also considered to be nutritious in terms of providing antioxidants. In other studies (e.g. Ickowitz et al. [41], the nutritional importance of wild foods could be especially high if they are consumed in sufficient quantities to compensate for shortfalls in agricultural production or imports. The consumption of edible wild fruits was also linked to improved diets. A key informant from the Department of Natural Resources Management in Mbire noted that:

I think I have seen this somewhere that dietary diversity is kind of changed, because the normal food that they usually eat, is not available anymore, so they go searching for other types of food or forest edible food, and what you get is more diverse now, instead of the less diversity that they had before, even though overall you know, high energy food consumption, micro nutrients and macro nutrients have gone down. I think it is quite a paradox, but the diversity is a bit higher…

Local households in Southern Africa make extensive use of wild edible fruits to sustain their livelihoods during crop failure. This is because most wild fruits are known to be nutritionally rich and high in vitamins and micronutrients [36]. According to Dube and Phiri [42], food security amongst rural communities in Zimbabwe has always been supported by the consumption of wild fruits especially during times of food crisis. The collection of wild foods is a commonly used risk-coping strategy by rural dwellers in developing countries [43]. According to Feyssa et al. [41], local people have good knowledge of wild fruit species, including their time of fruiting and ripening. This enables them to thoroughly plan and prepare for the future to ensure fruit availability. Some wild edible fruits are gathered, preserved, stored, and consumed some weeks or months after gathering [36].

#### 6.2.2 Psychosocial health

In terms of psychosocial health, varied experiences were shared. For the positive psychosocial health indicators, respondents who embarked on the adaptation measures were asked about any positive effect such actions had on their well-being over the previous four weeks. The relevant positive effects included: A sense of control over my life despite the challenge; Excitement at the opportunity to explore a new way of life; Pride in finding a new solution during adversity; and Grateful for having a viable option. From the qualitative data, one school headmaster articulated positive psychosocial health effect as:

Distribution of drought tolerant seeds and cash to vulnerable children help children not to feel out of place. They will be visualising themselves as people who own something in their life from the farming activities and the donations they receive from the donor. So it's something that boosts them socially and maybe economically…During the school feeding times, school attendance improves and if it ends we start experiencing drop outs. ….Food issues boost children's intelligence as well as class participation.

However, negative aspects were also exposed. The growing of drought tolerant crops requires inputs, which are not readily available (seeds not available in local shops,) and also income to purchase (if not given under the government or donor schemes). This induces worry and stress. The crops also do not fetch a desirable price at the market. Hence it was noted by one key informant that “*Family members are resorting to drunkenness in order to deal with financial and family challenges*” (Mental Health Nurse). A natural resources management expert in Mbire also added that:

The moment that farmers notice that they do not have enough food for the family, they end up being stressed, diseases such as high blood pressures increase, though we do not have real statistics, maybe you can get them from the health department.

### 6.3 Challenges in undertaking adaptation strategies

The issue of labour emerged prominently in the qualitative findings. For instance, some adaptation actions like *Pfumvudza* (climate smart agriculture), where growing of drought resistant crops is also promoted, are labour intensive, increasing work load particularly on women thereby affecting their physical and mental health*.* A prominent researcher at one tertiary institution highlighted that “*there is a need for development of appropriate technologies that helps these small farmers in terms of harvesting or processing, these small grains*”. Other challenges cited by Ngumbi [42] include that unlike traditional seeds, drought tolerant seeds have to be bought every year - though drought tolerant crops produce seeds, they lose some of their drought protection capacities; so farmers are encouraged to buy new seeds, not save them from the previous harvest. This puts financial strain on them and farmers are afraid of being locked in this cycle of financial obligation. Hence, due to financial challenges to get inputs, support in Mbire has come from other actors, for instance, Help Germany, to increase sorghum production, sunflower varieties and sesame (known as *runinga* or *chitohwe* in vernacular language). The Department of Social Development also distributes seeds and inputs for drought resistant crops such as cowpeas and sorghum to vulnerable families. Another challenge linked to output markets is that drought resistant crops are sold at low prices hence leaving families without enough funds for family survival. Lack of post-harvest management knowledge on other protein products such as beans and cowpeas only guarantees the provision of protein for a limited period of time which is a problem in terms of dietary diversity. It was also argued that for *Pfumvudza*, it seems the focus is on increasing production rather than on the nutritional value of crops whilst some farmers still do not have the mindset of accepting drought tolerant crops.

It also emerged that opening up of new agricultural land for drought resistant crops has resulted in human and wildlife conflicts as human beings are clearing animal habitats for agricultural production.

“People are clearing new settlements and new agriculture land because they want places to stay. Partly because water resources and other places that used to be pools, there is serious. Much of our water reservoirs doesn't keep water for the whole year like it used to do, so in many times there is so much human and wildlife conflicts especially when humans are now sharing resources with animals”

In terms of consumption of wild fruits and vegetables, conflict in access to resources emerged as a huge challenge. Harvesting of wild edible foods exposes people to surveillance and harassment from regulators such as the Environmental Management Agency, Forestry Department and the Police. Data shows that the main challenges for the households that consume wild fruits, insects and vegetables in Mbire District was conflict in harvesting or accessing the wild food resources which was cited by a key informant. This was followed by those who cited labour and declining wild food resources. It was also noted that limited knowledge on the preparation of wild fruits and vegetables led to adverse health effects. One village head highlighted that “*If not cooked properly, some of the wild fruits can be potentially poisonous e.g. katunguru fruit. It should be thoroughly boiled before consumption*”. In other studies, it has been noted that their consumption is intimately attached to location and culture, and their nutritional values are not known to many [44].

Regarding the use of rainfall and drought prediction indicators, communities still have diverse and at times conflicting and competing views. Working in similar communities in Zimbabwe, Mtambanengwe et al. [38] also reported that farmers have a varied understanding and belief of flora and fauna that best predict rainfall patterns and droughts. Some key informants noted that IKS is fragmented and how stakeholders are promoting the use of IK in adaptation is also not well articulated. IKS are not really embedded in the day to day work of the various stakeholders in dealing with small-holder farming communities. There were also concerns that rainfall and drought prediction knowledge was becoming extinct. A university researcher noted that:

…Traditionally people have these ways of forecasting weather. You find there are a lot of *Mazhanje* for example….It means it is not going to rain for example, so I think to some extent there this traditional climate smart knowledge that is out there, we are not actually documenting and I am afraid it is going to die natural death…

### 6.4 Opportunities in undertaking adaptation strategies

Regarding growing of drought tolerant crops, financial support to get inputs has mostly come from other external actors, for instance, Help Germany and ADRA, to increase sorghum production, sunflower varieties and sesame. The Department of Social Development also distributes seeds and inputs for drought resistant crops such as cowpeas and sorghum to vulnerable families. Opportunities to access inputs also emerge from various entities. A key informant from Social Services Department narrated that:

They participate in the Presidential Input Scheme where the vulnerable from social welfare benefit on one end while Agricultural Extension Department distributes to general farmers under the *Pfumvudza* (climate smart agriculture) program.

Networks and Partnerships also emerged as central for local government to be able to promote growing of drought tolerant crops. For example, the Rural District Council had partnered with the Agricultural Extension Department and the central government to encourage farmers to produce small grains and practice conservative farming. One local school has partnered with World Vision to distribute maize seed and drought resistant seed varieties to vulnerable children. In addition, *“Help Germany has promoted the preservation of the nutrition in vegetables by providing solar driers and about five wards benefited from the scheme”* said one district level key informant*.* Another key informant from the natural resources management in Mbire narrated that:

To also reduce human-wildlife conflict associated with opening new farmlands for drought tolerant crops, the Zimbabwe Agriculture Trust has been promoting set up of beehives in trees along traditional elephant routes to prevent elephants to come closer to human settlements.

The main opportunities arising from consuming wild fruits, insects and vegetables were sharing indigenous knowledge (which was cited by 38 % of households), followed by social cohesion or network in the community (cited by 22 % of the households). Narratives from key informants also indicated the importance of networks and partnerships. Involvement of non-governmental organizations and government entities towards initiating value addition projects within the communities (e.g. changing baobab fruits into grounded powder for porridge making, *masawu* fruit to jam – locally) were cited. It was noted that farmers are trained on value addition of indigenous fruits by SAFIRE organization and they are encouraged to process and package indigenous products such as *masawu* jam and *mawuyu* so as to market these products at higher prices.

## 7 Sustainability and scaling up

The majority of community members indicated that they would continue with the strategies, irrespective of the challenges they are facing and the inability to realize full nutritional and psycho-social benefits. However, the continued use of the strategies is confronted by a number of challenges and is based on a number of factors including support to gain access to inputs for drought tolerant seeds. It was also noted that there are challenges with the paucity of data on nutrition which affects sustainability considering that adaptations are driven by institutions. One University of Zimbabwe academic/researcher said that:

Agricultural extension department was also involved but the challenge with the donor driven activities was what happens after the end of the project you find everything that just ends there, but with the government department being involved it is easier to manage the strategies. Other national level key informants noted the need to commit adequate financial resources to current adaptation actions and incorporate the production of nutritional value crops in climate smart programmes like *Pfumvudza* for sustainability. Training of farmers also emerged as key in promoting sustainability so that the farmers use the knowledge in the absence of local or external support on how to grow DTC to maximize impacts and value addition to get the most value in consuming wild fruits.

Scaling up and adaptability of findings from this research is one of the innovations that the research sought to promote. Therefore, in general, we propose:

• Setting-up of guidelines on a routine/periodic evaluation of the impacts of adaptation actions in Zimbabwe – (through the Ministry of Environment/Climate Change Management Department) in conjunction with relevant state and non-state actors.
• Embedding aspects which could assist in assessing the positive and negative impacts
• of adaptation actions within regular surveys, such as the ZIMVAC survey.
• Addressing the challenges of coordination between state and non-state entities focused on similar activities so as to maximise impact.
• Reducing donor syndrome and empower local people to be able to produce their own food without relying on seed donations.
• Irrigation development in areas closer to rivers such as Angwa.
• Creating strong partnerships with other departments and nongovernmental organizations to support farmers in their strategies.
• Strengthening the linkages between adaptation strategies for food security and nutrition security (especially *Pfumvudza*).
• Setting up Community Assessment structures at ward, village and district levels for effective information dissemination.
• More research on nutritional benefits of drought tolerant crops is still required.
• Scale up consumption research studies on consumption of small grains and wild foods

### 7.1 Regarding drought tolerant crops

• Encouraging increase of small grain varieties production such as sorghum, millet, and sesame to ensure that there is food security even at household levels.
• Scale up research studies on consumption of small grains, wild foods, and wild fruits.
• There is need for appropriate food processing so that the nutritional benefits of small grains and traditional forest foods are realized.

### 7.2 Use of local drought and rainfall indicators

• There is need for a hybrid approach to strengthen linkages between IKS of climate prediction and modern scientific forecasts.
• IKS knowledge is fragmented and how stakeholders are promoting the use of IKs in adaptation is also not well articulated. IKS should be embedded in the day to day work of the various state and non-state stakeholders in dealing with smallholder farming communities.
• State and non-state institutions should push for the extensive documentation of IKS in communities.

### 7.3 From the perspective of psycho-social health

• Recognition of psychosocial health as a major component of adaptation action – in fact, psychosocial health has not been accorded much recognition in climate discourse in Zimbabwe. For instance, the Mental Health Unit of the Ministry of Health has not mainstreamed psychosocial health in its disaster responses including adaptation and recovery. Therefore some entry points for psychosocial health into climate interventions should include:
• Robust research on the nexus between climate change, adaptation and psychosocial health.
• Inter-departmental collaborations at government level, e.g. Food and Nutrition Council, Department of Mental Health, and Climate Change Management Department.
• Incorporation of psychosocial health into extension programs.
• Community awareness raising on the psychosocial health impacts of climate change an adaptation

## 8 Conclusion

There is a nexus between climate change and health. In particular, climate change adaptation is not a linear process and its linkages to health outcomes is complex. The findings in this study reveal that rural communities have the capacity to adapt but their strategies produce diverse outcomes. In general, outcomes are not given attention especially in emerging and still understudied areas such as nutritional and psychosocial health as well as marginalised communities. In this paper, it is poignant that there are some positive nutritional and psychosocial health benefits emerging from the two strategies under consideration. There are also negative aspects. However, we conclude that this study has opened new knowledge frontiers where adaptation studies need to move beyond either identifying the strategies or categorizing them as bad or good but delve deeper into understanding outcomes of strategies in domains that are still under-studied - nutritional and psychosocial health. This study has been more of a learning process aimed at learning from what exists in rural marginalised communities and identifying ways to adapt and upscale the results, and at the same time showing everyday practices and experiences of people with health outcomes of adaptation.

## CRediT authorship contribution statement

**Sandra Bhatasara:** Writing – original draft, Formal analysis, Investigation, Methodology, Writing – review & editing. **Chijoke Nwosu:** Formal analysis, Investigation. **Lesley Macheka:** Formal analysis, Writing – review & editing. **Admire M. Nyamwanza:** Formal analysis, Funding acquisition, Resources.

## Declaration of competing interest

The authors declare that they have no known competing financial interests or personal relationships that could have appeared to influence the work reported in this paper.
